# Problem-based learning in traditional Chinese medicine education: current applications and future directions

**DOI:** 10.3389/fmed.2026.1755513

**Published:** 2026-04-13

**Authors:** Naihan Jiang, Shuyan Li

**Affiliations:** Traditional Chinese Medicine (Zhongjing) School, Henan University of Chinese Medicine, Zhengzhou, China

**Keywords:** curriculum implementation, educational reform, learning outcome evaluation, problem-based learning, traditional Chinese medicine education

## Abstract

Problem-Based Learning (PBL), a student-centered and problem-oriented instructional approach, has increasingly been introduced into Traditional Chinese Medicine (TCM) education. This structured integrative review synthesizes existing empirical studies examining the application of PBL across foundational, clinical, and classical literature courses in TCM curricula. Available evidence suggests that PBL may be associated with improvements in student engagement, case-based reasoning, and collaborative learning within specific educational contexts. However, the current literature is largely composed of single-institution, quasi-experimental studies with heterogeneous outcome measures and limited long-term evaluation. In addition to summarizing reported benefits, this review identifies persistent challenges, including insufficient case standardization, variability in faculty facilitation, and misalignment between instructional design and assessment systems. Based on these findings, several directions for future research and curriculum development are outlined. Overall, while PBL represents a potentially valuable complementary instructional strategy in TCM education, further rigorous and standardized investigations are required to clarify its sustained educational impact.

## 1 Introduction

With the continuous advancement of global medical education reform, traditional teacher-centered and lecture-based instructional models are gradually giving way to approaches that emphasize student engagement and the development of clinical competencies. Among these, Problem-Based Learning (PBL) has been widely adopted in various medical education systems as a learner-centered, problem-driven pedagogical strategy (1). PBL has demonstrated significant educational advantages, including enhanced learning motivation, improved participation, and the promotion of clinical reasoning, critical thinking, and teamwork—competencies that are increasingly valued in modern medical training (2).

Against this backdrop, Traditional Chinese Medicine (TCM) education faces an urgent need for pedagogical transformation. Currently, TCM teaching is often criticized for a disconnect between theory and practice, outdated curricula, low student interest, and limited classroom interaction (3). TCM has a unique theoretical system emphasizing holistic thinking, syndrome differentiation, and experiential inheritance. Traditional didactic methods are often insufficient to convey its complexity and internal logic, thus impeding students’ comprehensive understanding and clinical application abilities (4).

In recent years, several TCM institutions in China and abroad have initiated efforts to incorporate PBL into their educational practices, particularly within basic theory, formula science, diagnostics, clinical courses, and classical text studies. While preliminary studies have reported positive outcomes (5), the current literature remains fragmented and lacks systematic synthesis and evaluation. Especially from the perspective of a full-course TCM curriculum, questions remain regarding the compatibility of PBL with TCM pedagogy, its implementation pathways, assessment mechanisms, and future development directions.

Therefore, this review aims to comprehensively examine the current application status of problem-based learning (PBL) in various modules of traditional Chinese medicine education, integrate existing empirical studies, deeply explore its compatibility with the concepts of traditional Chinese medicine education, summarize its teaching advantages and practical challenges, conduct a critical analysis of the opportunities and constraints for applying PBL in traditional Chinese medicine courses, and look forward to its future development directions in cross-disciplinary integration and digital transformation.

## 2 Methods

### 2.1 Search strategy

A systematic literature search was performed in the following electronic databases: PubMed, Web of Science, China National Knowledge Infrastructure (CNKI), Wanfang Data, and Weipu Chinese Journal Database. The search yielded a total of 187 records across all databases. The search covered the period from January 2000 to October 2025, reflecting the emergence and growth of PBL applications in TCM education. The search strategy combined controlled vocabulary terms and free-text keywords. The following Boolean combinations were used (adapted as appropriate for each database):

(“Problem-Based Learning” OR “PBL”) AND (“Traditional Chinese Medicine” OR “TCM” OR “Chinese Medicine Education”)

(“PBL teaching” OR “problem-oriented learning”) AND (“TCM course” OR “TCM curriculum” OR “Chinese medicine education”)

Both English- and Chinese-language publications were included. Reference lists of eligible articles were manually screened to identify additional relevant studies.

### 2.2 Inclusion and exclusion criteria

#### 2.2.1 Inclusion criteria

(1) Focused on the application of PBL in TCM education (including foundational, clinical, and classical literature courses); (2) Reported empirical data on educational outcomes (e.g., learning engagement, examination performance, clinical reasoning ability, communication skills).

#### 2.2.2 Exclusion criteria

(1) Were opinion pieces, commentaries, editorials, or purely theoretical discussions without empirical data; (2) Focused solely on PBL in Western medicine without TCM relevance; (3) Lacked sufficient information regarding study design or outcomes.

### 2.3 Research screening and data extraction

All the retrieved records were imported into the reference management software and duplicate items were removed. After deduplication, 146 articles remained for further screening. Two reviewers independently reviewed the titles and abstracts, and evaluated the eligibility of the studies according to the preset criteria. During this stage, a total of 96 papers that were unrelated to traditional Chinese medicine education and did not involve problem-based learning or lacked empirical data were excluded. Subsequently, a full-text review was conducted on the 50 retained articles. Based on their relevance to the review topic, the clarity of the methodology, and the overall academic representativeness, a total of 26 studies met the inclusion criteria and were included in the qualitative comprehensive analysis. In case of disagreement, it was resolved through discussion or consultation with a third reviewer. Data were extracted using standardized tables, including: authors, year, institution, course type, research design, sample size, details of PBL implementation, outcome measurement indicators, and main findings.

### 2.4 Quality assessment

Given the diversity of study designs, methodological quality was appraised using criteria adapted from the Mixed Methods Appraisal Tool (MMAT). The assessment considered: Clarity of study objectives; Appropriateness of study design; Description of intervention; Transparency of outcome measurement; Presence of comparison group; Statistical reporting.

### 2.5 Data synthesis

Due to heterogeneity in study design, course context, and outcome measures, a meta-analysis was not feasible. Therefore, the 26 included studies were analyzed using a narrative synthesis approach. Instead, a narrative synthesis approach was adopted. Findings were organized into three analytical domains: (1) Applications of PBL across different curriculum modules (foundational, clinical, classical literature); Effects on learning engagement and motivation; (2) Effects on clinical reasoning, academic performance, and collaborative competencies; (3) Patterns, consistencies, and methodological limitations across studies were critically examined to identify both convergent findings and evidence gaps.

## 3 Development of PBL in medical education

PBL was initially proposed by the Faculty of Medicine at McMaster University in Canada in the late 1960s as an alternative to the traditional lecture-based medical education. This educational approach has faced increasing criticism for overemphasizing memory and neglecting clinical reasoning and problem-solving abilities (1). The core concept of PBL emphasizes learning through real-world problems, group collaboration, and guided inquiry - marking a shift from teacher-centered knowledge transmission to learner-centered knowledge construction. In the 1970s and 1980s, PBL was adopted and further refined by institutions such as Maastricht University in the Netherlands (6), and subsequently implemented in medical schools in North America, Europe, and Australia (7). Reports from these institutions indicate that learners’ engagement, knowledge integration, and communication skills have improved, suggesting its effectiveness as an alternative approach (8).

In the early 21st century, PBL expanded beyond medicine into related health professions, including nursing, pharmacy, rehabilitation, and public health. It also gained a foothold in Asia, where mainland China began introducing PBL into medical school pilot programs around the year 2000. Initially implemented in basic science courses such as physiology, pathology, and immunology, PBL gradually extended into clinical clerkships and standardized patient-based teaching. Encouraging outcomes from these pilots prompted broader adoption and triggered interest in developing PBL strategies tailored to Chinese educational culture and institutional frameworks (9, 10).

**Figure 1:**
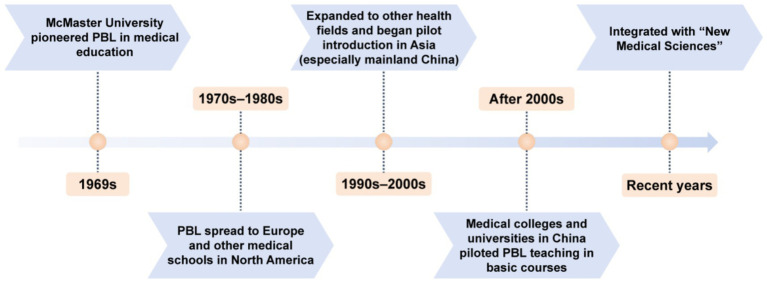
PBL development timeline.

The development of PBL from its inception in the 1960s to its global dissemination over the past decades reflects its adaptability and enduring relevance in health education (Figure 1). As China continues to reform medical education under the “New Medical Sciences” initiative, PBL offers promising potential for transforming TCM education—helping bridge the gap between theoretical knowledge and clinical application while fostering innovation in curriculum design and instructional methods.

Although PBL has been widely adopted, given the unique epistemological framework and curriculum structure of traditional Chinese medicine, the application effect of PBL remains a topic of continuous discussion. Although many research reports claim that this method can enhance students’ satisfaction and clinical reasoning ability, systematic reviews indicate that the results vary. Moreover, the quality of implementation, teacher training, and the matching degree with the assessment system seem to have a significant impact on the reported effects. This highlights the necessity of structuring a comprehensive synthesis of existing empirical research specific to the context of traditional Chinese medicine, and this review is precisely carried out for this purpose.

## 4 Current challenges in traditional Chinese medicine education

The current traditional Chinese medicine education is constructed based on the main contents of basic theories, clinical practice and research on classic Chinese medical literature, forming a comprehensive and highly specialized knowledge system. Although its curriculum is relatively comprehensive, the current teaching mode is still mainly based on lecturing, which has triggered several structural problems and affected students’ learning outcomes and ability development.

In foundational courses such as Basic Theories of TCM, Formulas, and TCM Diagnostics, the dominant teaching method still relies heavily on rote instruction. Content is closely tied to textbooks, and classroom delivery lacks variety. As foundational components of TCM’s disciplinary knowledge system, theories such as Yin–Yang, Five Elements, and Zang–Fu organs (11, 12) are often memorized by students without sufficient opportunities to understand or internalize their underlying logic. Because these courses are not well integrated, students struggle to form a coherent understanding of the TCM knowledge system. For example, a student might memorize the composition of the herbal formula Ma Huang Tang (13) in a formulas course, yet remain unclear about when and how it is applied in clinical settings. The limited integration among various courses and the disconnection between theoretical knowledge and practical application further restrict the students’ ability to integrate concepts into a coherent diagnostic reasoning framework.

Clinical training modules, including Internal Medicine of TCM and Gynecology of TCM, aim to cultivate syndrome differentiation and treatment planning abilities. However, instructional practice in these courses is often constrained by insufficient case standardization and variability in teaching formats (14, 15). Case discussions may rely heavily on individual instructors’ experiences rather than structured reasoning pathways, and assessment methods remain predominantly written examinations. Consequently, opportunities for systematic development of clinical decision-making skills may be uneven.

TCM classical literature courses present additional pedagogical complexity. Texts such as Huangdi Neijing, Shanghan Lun, and Jingui Yaolue contain the core theories of traditional Chinese medicine, but they are highly challenging in terms of language and concepts (16, 17). Teaching usually focuses on the interpretation and annotation of the texts, while the emphasis on clinical context is relatively less. This teaching approach may reduce students’ engagement and hinder the integration of classical knowledge with contemporary clinical reasoning.

**Figure 2:**
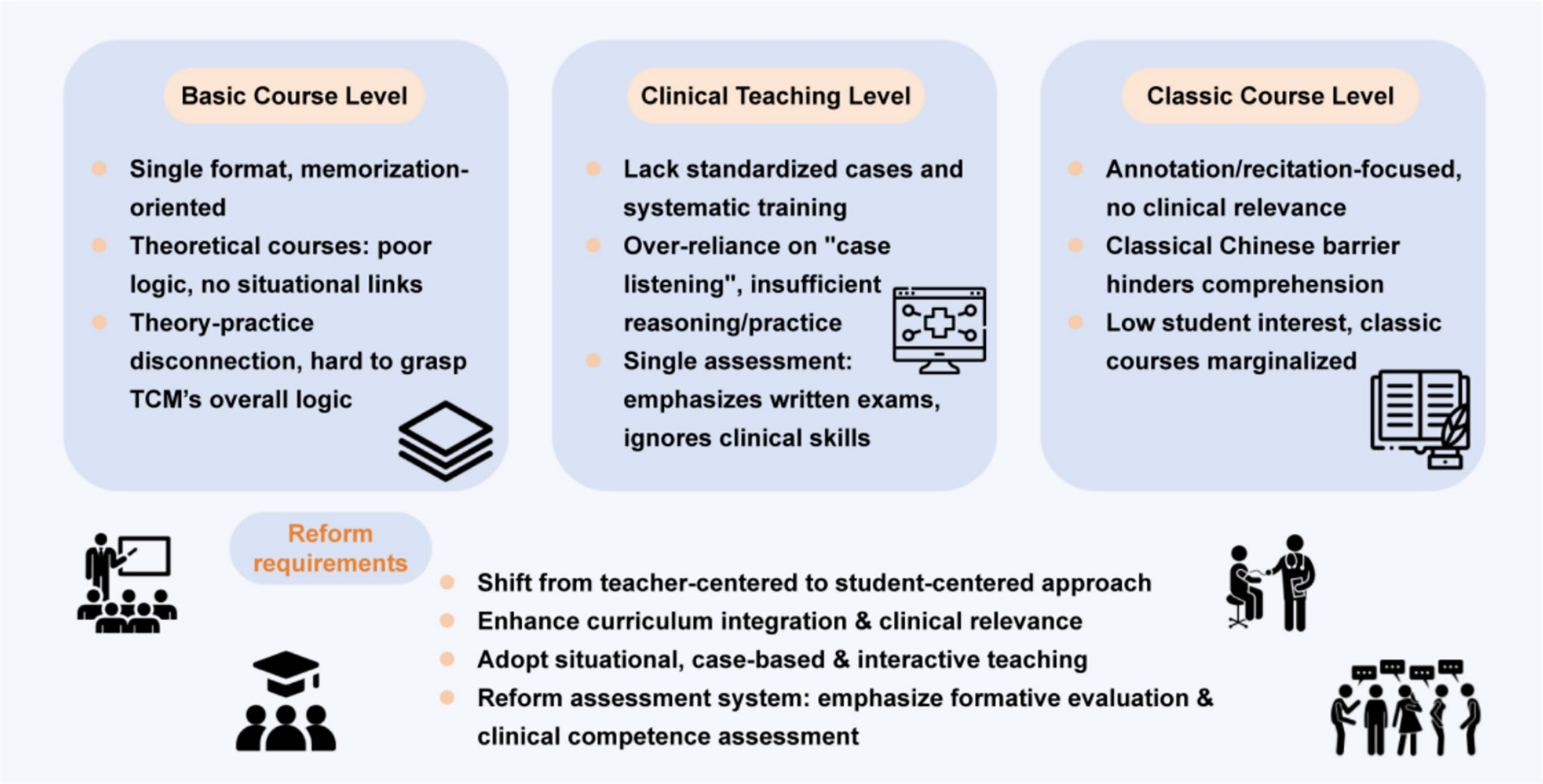
Framework of current issues and reform needs in TCM education.

Taken together, these challenges reflect a broader tension within TCM education: balancing the preservation of disciplinary tradition with the development of applied competencies. The need for more interactive, integrative, and competency-oriented instructional strategies has therefore been increasingly recognized. In this context, PBL has been introduced by some institutions as a potential complementary approach to address these structural gaps, particularly in fostering active learning and contextualized reasoning. Figure 2 presents the current issues and reform demands in traditional Chinese medicine education.

## 5 Applications of PBL in TCM courses

In response to structural challenges within TCM education, various institutions have explored the integration of PBL across foundational, clinical, and classical literature modules. Although implementation formats vary, existing studies reveal several common patterns in instructional design.

In foundational subjects such as Basic Theories of TCM, Diagnostics, and Formulas, PBL is primarily employed to facilitate the integration of abstract theoretical principles with clinical reasoning processes. Rather than presenting conceptual content in isolation, instructors design scenario-based problems that require students to interpret symptoms, analyze tongue and pulse data, and propose syndrome differentiation hypotheses (15, 18, 19). This approach shifts learning from memorization of theoretical constructs toward active construction of diagnostic logic. Students are encouraged to articulate reasoning pathways linking pathogenesis, syndrome identification, and treatment principles. In this context, the role of the instructor transitions from knowledge transmitter to discussion facilitator, guiding inquiry without providing immediate solutions.

In clinical courses, including Internal Medicine of TCM, Surgery of TCM, and Gynecology of TCM, PBL is commonly integrated with case-based tasks designed to simulate authentic diagnostic and therapeutic processes (20–22). Students are typically required to analyze patient histories, interpret findings from the four diagnostic methods, formulate syndrome differentiation strategies, and propose treatment plans within collaborative groups. Compared with foundational courses, clinical PBL implementations tend to focus more explicitly on constructing complete reasoning chains-from etiology and pathogenesis to prescription selection. Some programs have incorporated simulated outpatient scenarios or structured consultation procedures to approximate real-world clinical decision-making environments (22). Despite variability in design complexity, a shared characteristic of clinical PBL is the emphasis on contextualized reasoning and peer discussion, intended to mirror the dynamic nature of TCM diagnostic practice.

The application of PBL in classical TCM literature courses has emerged more gradually but reflects distinctive pedagogical experimentation. Instead of relying solely on textual annotation and translation, instructors have introduced classical excerpts or historical medical cases as inquiry triggers (23, 24). Students are tasked with interpreting original passages, reconstructing underlying diagnostic logic, and relating classical reasoning to contemporary clinical scenarios. This format seeks to transform passive textual study into active analytical engagement. In some implementations, digital resources and online databases are incorporated to support independent exploration of classical materials (24).

**Figure 3:**
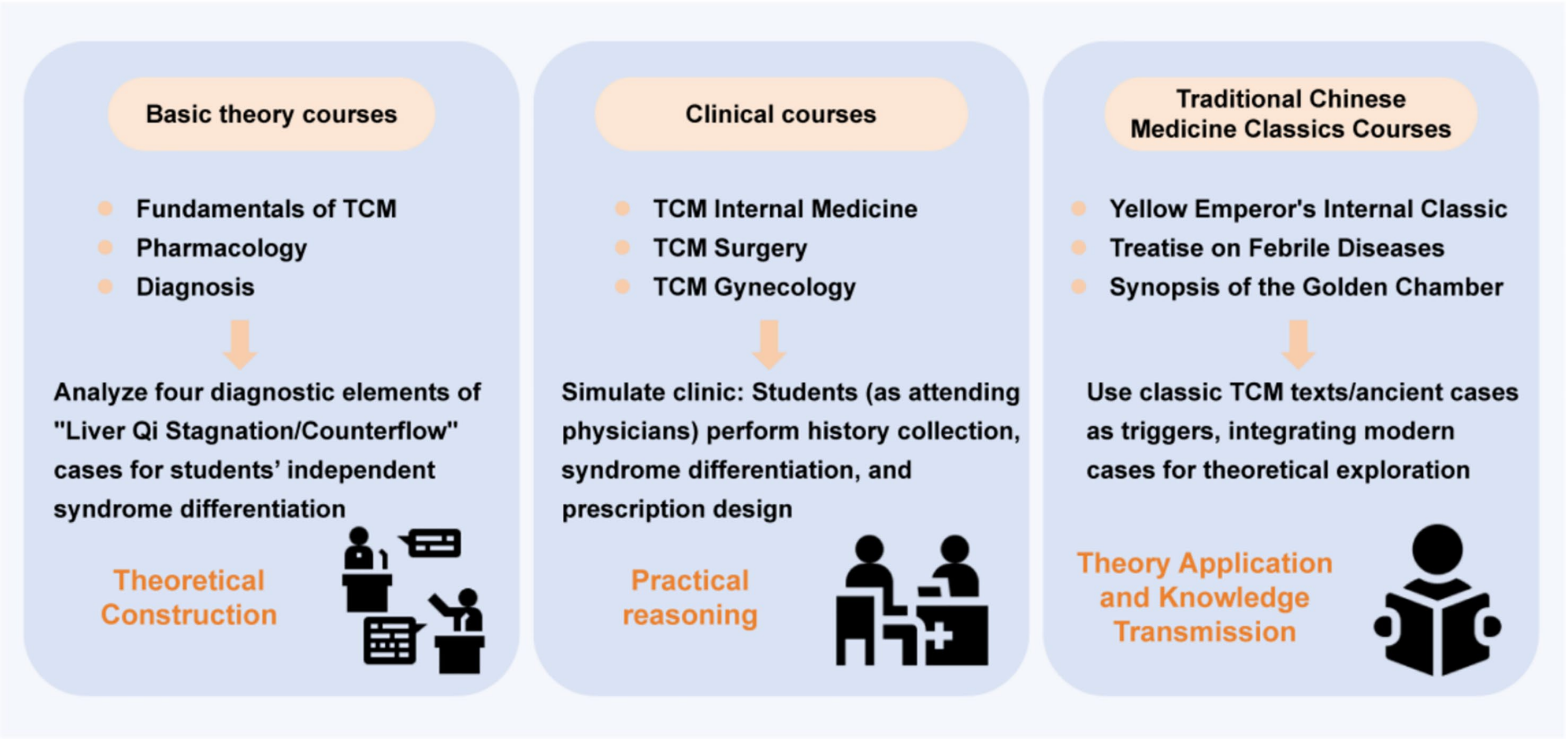
Scope of PBL applications in TCM education.

Across course types, several recurring implementation patterns can be identified: small-group discussion as the primary instructional unit; problem scenarios grounded in authentic or semi-authentic clinical contexts; facilitator-guided inquiry rather than direct instruction; emphasis on linking theoretical constructs with diagnostic reasoning. Most documented implementations are reported in single-institution studies and frequently describe pilot reforms rather than fully institutionalized curricular redesign. The application of PBL in traditional Chinese medicine education demonstrates a high degree of alignment between teaching design and course characteristics (Figure 3). Although the scope of these measures is relatively limited and often confined to pilot projects, they showcase attempts to apply the PBL principles to the cognitive features of traditional Chinese medicine classical education.

## 6 Evidence evaluation and critical perspectives

Most included studies adopted quasi-experimental or comparative cohort designs, typically contrasting PBL-oriented instruction with traditional lecture-based formats within a single course module. No multi-center randomized controlled trials were identified. Sample sizes were generally modest, and implementation contexts were institution-specific.

Across studies, improvements in student engagement were among the most consistently reported findings. Increased classroom participation, self-directed learning behavior, and perceived learning motivation were frequently observed following PBL implementation. However, these outcomes were predominantly measured using locally developed questionnaires or post-course surveys, with limited reporting of psychometric validation. With respect to clinical reasoning and academic performance, several studies reported higher case analysis scores or improved diagnostic reasoning in PBL cohorts. Nevertheless, outcome measures varied substantially across studies, ranging from written examinations to instructor-rated presentations. Objective structured clinical examinations (OSCE) and standardized reasoning rubrics were rarely employed. Furthermore, most evaluations focused on short-term post-intervention performance, with little evidence regarding long-term retention or transferability of skills. Reported gains in teamwork and communication competencies were similarly based largely on self-report or instructor observation. Few studies utilized validated behavioral assessment tools. Taken together, existing findings suggest that PBL may be associated with positive short-term educational outcomes in specific TCM course contexts. However, methodological heterogeneity and limited standardization constrain definitive interpretation.

PBL is not the only active learning model explored in TCM education. Case-Based Learning (CBL), Team-Based Learning (TBL), and simulation-based instruction have also been piloted in various curricular settings. Compared with PBL, CBL often provides more structured guidance and predefined learning objectives, potentially reducing cognitive overload while preserving contextual learning. TBL emphasizes pre-class preparation and structured team application, offering greater control over content coverage. Simulation-based approaches facilitate skill rehearsal but may require substantial institutional resources. Evidence from broader health professions education indicates that the effectiveness of PBL is context-dependent. While it has been associated with enhanced learner satisfaction and reasoning processes, its superiority in knowledge acquisition remains inconsistent. Some studies report comparable or only marginal differences relative to traditional instruction, and implementation quality appears to significantly influence outcomes. These findings suggest that PBL should be considered one component within a broader pedagogical ecosystem rather than a universally superior alternative.

Overall, the current evidence base regarding PBL in TCM education can be characterized as exploratory and context-specific. Reported benefits are generally short-term and derived from small-scale, single-institution studies. The absence of multi-center designs, standardized outcome frameworks, and longitudinal follow-up limits generalizability. Accordingly, PBL in TCM education should presently be regarded as a promising but not yet conclusively validated instructional strategy. Future studies should design multi-center randomized controlled trials with matched control groups or well-designed quasi-experimental studies, and use validated tools (such as standardized clinical reasoning scoring criteria and objective performance indicators) to conduct longitudinal evaluations of skill retention (6–12 months) and assess their transfer to clinical practice. They should also follow the CONSORT/STROBE guidelines to ensure the transparency and reproducibility of the reports.

## 7 Implementation challenges in the TCM context

Beyond methodological limitations in the evidence base, several structural and contextual factors constrain the broader implementation of PBL in TCM education.

First, the development of high-quality case resources presents a significant challenge. Effective PBL in TCM requires cases that accurately reflect syndrome differentiation logic, integration of the four diagnostic methods, and the epistemological characteristics of classical theory. Designing such cases demands both disciplinary expertise and pedagogical competence, and insufficient case depth may reduce the authenticity of inquiry-based learning.

Second, faculty readiness varies considerably. PBL requires instructors to assume facilitative roles, manage group dynamics, and design open-ended tasks. Many educators trained in traditional lecture-based systems may lack formal preparation in these instructional approaches. Inconsistent facilitation quality can therefore influence student experience and learning outcomes.

Third, alignment between PBL pedagogy and assessment systems remains incomplete. Traditional evaluation in TCM education frequently emphasizes theoretical recall through written examinations. Without multidimensional assessment frameworks capturing reasoning processes, collaboration, and applied competencies, the sustainability of PBL initiatives may be limited.

Finally, student adaptability must be considered. Learners accustomed to teacher-centered instruction may initially experience difficulty adjusting to self-directed inquiry and group-based reasoning tasks, particularly in educational environments where independent learning readiness is limited (8, 9). Without appropriate scaffolding, PBL may increase cognitive load rather than promote deeper integration.

Addressing these systemic factors is essential if PBL is to move beyond pilot-level experimentation toward sustainable curricular integration.

## 8 Future directions for PBL in TCM education

Although preliminary evidence suggests that PBL may support engagement and case-based reasoning in TCM education, its broader and sustained implementation requires structured development rather than isolated pilot initiatives. Based on the synthesized findings and identified challenges, several strategic directions merit consideration.

First, curriculum-level integration should move beyond single-course experimentation toward coherent vertical design. Rather than applying PBL sporadically, institutions may consider adopting a staged or spiral framework in which problem complexity increases progressively from foundational to clinical and classical modules. Such alignment may facilitate cumulative development of diagnostic reasoning while preserving curricular continuity.

Second, faculty development remains essential for ensuring instructional quality. Effective PBL requires facilitation skills, case design competence, and alignment between inquiry tasks and assessment criteria. Structured training programs, peer observation systems, and standardized facilitation guidelines may help reduce variability in implementation and strengthen instructional consistency.

Third, case standardization and resource development should be prioritized. High-quality PBL in TCM depends on authentic cases that reflect syndrome differentiation logic, integration of the four diagnostic methods, and connections to classical theory. Developing structured case repositories within institutions—or through collaborative networks—may enhance sustainability and reduce redundancy while preserving disciplinary specificity.

Fourth, assessment reform should accompany pedagogical innovation. Without evaluation systems that capture clinical reasoning processes, collaboration, and applied competencies, the long-term impact of PBL may remain difficult to demonstrate. Incorporating structured reasoning rubrics, formative feedback mechanisms, and, where feasible, objective clinical assessments may improve alignment between instructional goals and outcome measurement.

Finally, adaptation to the epistemological characteristics of TCM remains critical. Rather than directly transplanting Western PBL models, future efforts should explore context-sensitive modifications that respect the interpretive, pattern-based, and classical foundations of TCM reasoning.

Overall, advancing PBL in TCM education will require incremental, evidence-informed refinement rather than wholesale replacement of existing teaching models. Continued comparative research and standardized outcome evaluation are necessary to clarify its long-term educational value.

## 9 Conclusion

This review summarizes the existing evidence regarding PBL in traditional Chinese medicine education. The research results indicate that PBL can enhance students’ participation, clinical reasoning ability, and collaborative learning skills, and shows performance in basic courses, clinical courses, and classic courses. However, the current evidence base is still relatively weak in methodology - mainly consisting of small-scale, single-center studies, and lacking objective assessment indicators and long-term evaluations.

Therefore, the application of PBL in traditional Chinese medicine education at present should be regarded as a promising but not yet fully validated teaching method. Its effectiveness seems to depend on systematic implementation, appropriate teacher training, and coordination with the assessment system. In the future, more rigorous and standardized designs are needed for research to clarify its continuous educational impact in traditional Chinese medicine courses and its broader applicability.
